# Textile-Based, Interdigital, Capacitive, Soft-Strain Sensor for Wearable Applications

**DOI:** 10.3390/ma11050768

**Published:** 2018-05-10

**Authors:** Ozgur Atalay

**Affiliations:** Faculty of Textile Technologies and Design, Istanbul Technical University, İnönü Caddesi, No. 65 Gümüssuyu, 34437 Beyoğlu/Istanbul, Turkey; atalayoz@itu.edu.tr; Tel.: +90-212-293-13-00

**Keywords:** wearable electronics, soft strain sensors, capacitive sensors, electronic textiles, conductive fabrics, silicone elastomer

## Abstract

The electronic textile area has gained considerable attention due to its implementation of wearable devices, and soft sensors are the main components of these systems. In this paper, a new sensor design is presented to create stretchable, capacitance-based strain sensors for human motion tracking. This involves the use of stretchable, conductive-knit fabric within the silicone elastomer matrix, as interdigitated electrodes. While conductive fabric creates a secure conductive network for electrodes, a silicone-based matrix provides encapsulation and dimensional-stability to the structure. During the benchtop characterization, sensors show linear output, i.e., R^2^ = 0.997, with high response time, i.e., 50 ms, and high resolution, i.e., 1.36%. Finally, movement of the knee joint during the different scenarios was successfully recorded.

## 1. Introduction

Electronic textiles have recently gained considerable research interest due to their possible application opportunities in different areas, such as human motion monitoring [1,2,3], soft robotics [4,5], physiological monitoring [6,7,8], temperature measurement [9,10], and human–machine interfaces [11,12]. Electronic textiles are lightweight, flexible, and unobtrusive compared with their rigid counterparts, and their sensing function is the essential part of these structures. In general, sensors can be described as devices that detect a change in physical stimulus and translate it into a signal that can be measured or recorded [13]. Among these structures, soft, stretchable strain sensors are gaining attention due to the large application opportunity in the aforementioned areas.

Until now, two measurement technologies, i.e., resistive and capacitive technology, were mostly used to developed textile-based strain sensors. The measurement principle of the resistive method relies on a change in the electrical resistance value of the conductive structure upon applied strain [14]. Conductive structures could be either on the yarn [15] or fabric levels [16]. However, the latter is mostly preferred, due to its easy integration into everyday clothing. For this aim, different methodologies have been developed to create resistive sensors on a fabric level. Coating with conductive polymers and conductive polymer composites could be considered as a first method [17,18,19,20]. However, some previous studies showed that fabrics became stiffer in proportion to the increased level of conductive polymer application, due to the relatively high stiffness of the conductive polymer compared to the fabric structure [21]. Delamination of conductive materials from the fabric structure under repetitive loading conditions is also a critical problem for this type of sensor. As an alternative method to conductive coating, some researchers created conductive zones within the fabric structure by using fabric manufacturing techniques, to create resistive sensors. In this case, knitting technology offers use of conductive yarns during the fabric manufacturing process. For this aim, researchers knitted conductive yarns to form conductive fabrics, and such sensors rely predominantly on the natural structural elasticity of the knitted fabric to provide recovery after stress deformation [22,23,24]. However, this production method has some disadvantages, such as the limited elasticity of the structure, which offers a relatively small working range. As a solution to this problem, the elasticity level of the sensor is enhanced by knitting conductive yarns along with elastomeric yarns [25]. In this case, contact resistance between the knitted, conductive loops, and changes in fabric dimensions under applied strain are responsible for the resistance change of the fabric. Contact resistance dominates the total resistance under the initial strain, whereas length-related resistance determines the total resistance for increased strain; thus, this phenomenon makes the sensor usage difficult for practical applications due to complicated sensing behavior.

As an alternative method to resistive sensing technology, capacitive-based sensors offer some advantages over other systems, such as higher linearity, less hysteresis, and fast response time, which are important parameters when the sensors are intended to be used in real-life scenarios [26,27,28,29]. Until now, mostly silicone-based soft sensors have dominated research in this area, and a parallel plate capacitor arrangement was utilized to build the sensing structures. In these systems, a soft, dielectric layer, i.e., silicone elastomer, is sandwiched between conductive soft plates, and conductive elements are employed to create conformable electrodes in the sensing system. For this aim, nanowires [26], nanotubes [28], graphene [30], carbon black [31], and conductive fabrics [32] are used. Although various strategies have been developed to make these conductive materials stretchable within the sensing system, they are mostly prone to cracking and delamination over extended usage of the sensor. It should be noted that the parallel plate capacitor arrangement also responds to applied pressure changes. Therefore, when the sensor is indented to be used in real-life applications, obtaining signals that are only derived from strain becomes difficult.

To address the above-mentioned challenges, we introduce a sensing structure that combines conductive-knit textile and silicone elastomer. The conductive properties of the electrodes affect the overall performance of the capacitive sensor, and it is crucial to use electrodes with good conductive performance under repetitive loading. Therefore, the usage of knitted fabric enables continuous conductivity within the structure, as well as a secure conductive network. We also devised an interdigital arrangement for the capacitive sensor, which is a one-layer structure in which conductive, knitted fabric is placed in a comb-shaped arrangement. Thus, thinner structures can be achieved compared to the parallel plate capacitor arrangement, thereby creating more compliant and flexible sensors. In addition, due to its one-layer structure, this arrangement is only responsive to strain measurements. The following section describes the production methodology of the soft sensors, along with their testing methodology. The third section reports the results obtained from the experimental procedure and discussion of the electro-mechanical properties of the sensor.

## 2. Materials and Methods

### 2.1. Manufacturing Methodolgy of the Sensors

**Figure 1:**
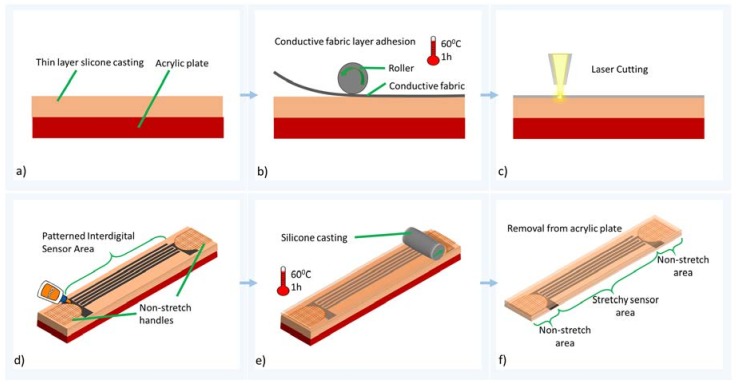
Schematic diagram of the manufacturing process. (**a**) Silicone casting on acrylic plate. (**b**) Adhering the conductive fabric to silicone elastomer. (**c**) Laser cutting of interdigitated shape. (**d**) Adding of non-stretch handles. (**e**) Top layer silicone casting and further curing. (**f**) Removal of the sensor from acrylic plate.

Figure 1 describes the manufacturing process of the stretchable strain sensor. Initially, a film of silicone elastomer (Ecoflex 30, Smooth-on, Macungie, PA, USA) is cast on an acrylic plate, using a thin film applicator, as shown in Figure 1a. Ecoflex 30 was chosen due to its superior properties for wearable applications, such as good stretchability, a high elongation limit (900%), and being non-toxic to the human body. Next, stretchable, conductive-knit fabric (Shieldex^®^ MedTex P130, Palmyra, NY, USA, highly conductive, with a surface resistivity of <1 ohm/sq.) adheres to the silicone solution. The silicone solution and conductive fabric structure is cured in an oven at 60 °C for one hour, as shown in Figure 1b. Thereafter, the cured structure on an acrylic plate is ready to be cut by laser. It is crucial that the cured structure is not removed from the acrylic plate at this stage. Herein, an interdigitated shape is engraved, using a laser (LS 6.60, Universal Laser Systems, Scottsdale, AZ, USA) at 40 W power and 5% speed setting. The machine setting is carefully optimized such that the interdigitated shape is carved out by burning the conductive fabric. Excess fabric parts around the interdigitated shape are removed from the structure by peeling them away.

**Figure 2:**
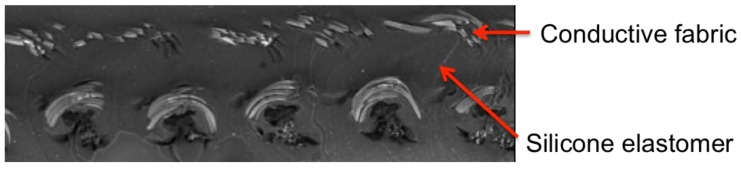
SEM image shows a cross-section of the conductive fabric, as well as the silicone encapsulation layer within the structure.

Thereafter, the non-stretchable, woven fabric is added as handles, and silicone elastomer is cast into the structure again. At this stage, silicone elastomer fills the areas between electrodes, forming the dielectric layers, and encapsulates the structure. Figure 2 shows penetration of silicone elastomer into the conductive fabric. As the last step, the whole system is further cured at 60 °C for one hour, and the thickness of the whole structure measures 1 mm. This type of design has some advantages over the parallel plate arrangement, as such sensors are able to accommodate surfaces of irregular shape easily.

Five samples are produced through this manufacturing methodology and consistent baseline capacitance values are obtained (17 pF ± 0.15). Thus, this methodology can produce sensors with nearly identical properties.

### 2.2. Electromechanical Characterization of the Sensor

In order to characterize the electromechanical properties of the soft sensors, an electromechanical tester (Instron 5544A, Norwood, MA, USA) was used. During the electromechanical testing, sensors were placed on the top and bottom clamps of the tensile tester. The bottom clamp was fixed while the top clamp was displaced at a predetermined speed. The capacitance values of the sensors were recorded with a capacitance meter. The force, extension, and capacitance data were synchronously logged through a common I/O interface, (BNC-2111, National Instruments Corp., Austin, TX, USA). A 50% level of strain was applied during the characterization of sensors, as this strain level meets the requirements of detecting posture and movement of the human body. Through this experimental set-up, the gauge factor, response time, and resolution of sensors were calculated. In addition to these parameters, drift behavior was also determined.

## 3. Results and Discussion

### 3.1. Electro-Mechanical Theory

**Figure 3:**
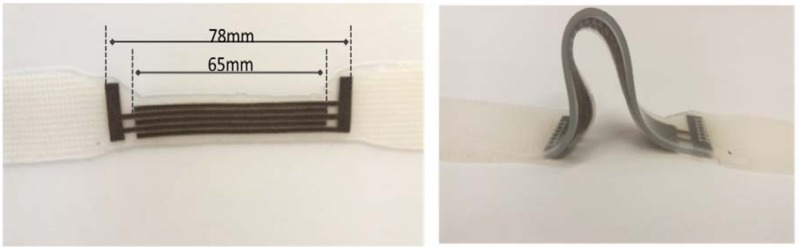
The real images of the sensor.

The sensor can be treated as a simple, interdigitated, capacitive sensor, and capacitance of the interdigital capacitive sensors is described as shown in Equation (1).

(1) $$C_{L}=\left(n-1\right)\frac{\varepsilon \;.\varepsilon r\;Wt}{d},$$

where C_L_ represents the capacitance value; *ε* and *εr* are permittivities associated with free space and the dielectric layer, respectively; *W* and *d* are the overlapped length of electrodes and the distance separating the two electrodes, respectively; *t* is the thickness of the conductive electrode; and n is the number of interdigital electrodes within the structure. Figure 3 shows the sensor’s real images.

**Figure 4:**
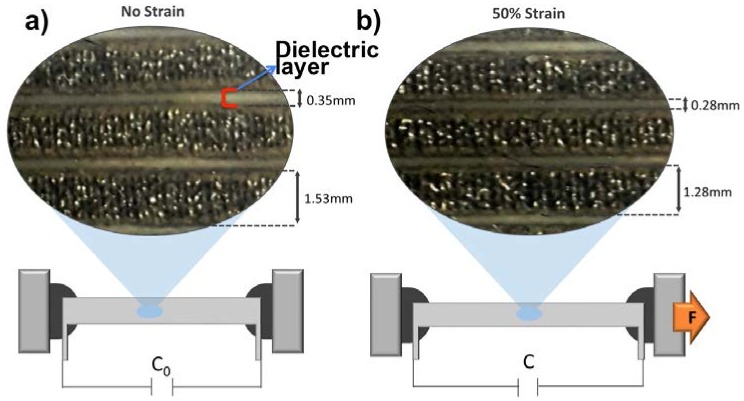
Magnified images of the sensor under (**a**) 0% strain and (**b**) 50% strain.

In this capacitive sensor design, *W* is equal to 65 mm, *t* is equal to 0.45 mm, *d* is equal to 0.35 mm, and n is equal to 5, as shown in Figure 3 and Figure 4. However, when strain is applied to the sensor, the distance between the electrodes decreases, i.e., 0.28 mm, due to Poisson’s ratio (Ecoflex poisson’s ratio is around 0.5) effect, which means the width of the silicone elastomer decreases when the structure is stretched. On the other hand, effective overlapped length between the electrodes increases, i.e., 97.5 mm, due to the increasing length of the conductive, knitted electrodes upon the applied strain, and the thickness of the electrodes reduces to 0.35 mm. Thus, the combination of these effects results in an increase in the capacitance value of the sensor. Figure 4 shows the decreasing dielectric thickness under applied strain.

### 3.2. Electro-Mechanical Properties of the Conductive Fabric

**Figure 5:**
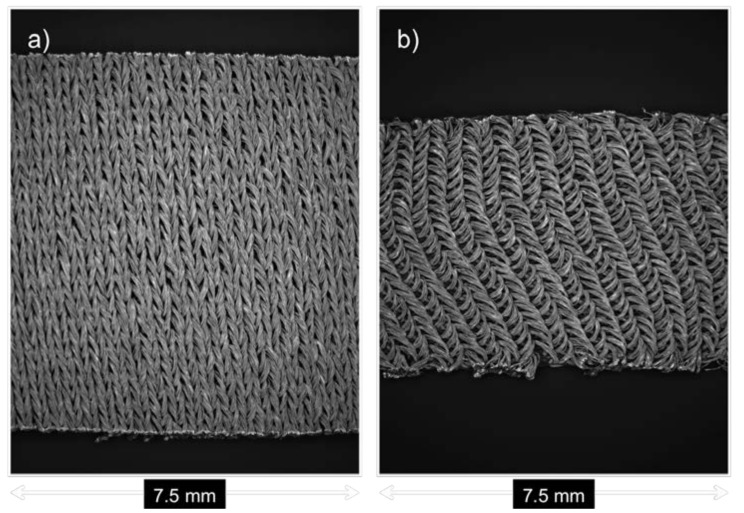
Magnified images show the fabric (**a**) at rest and (**b**) under applied strain.

The electrical properties of the conductive fabric under applied strain were also investigated, in order to observe the effect on sensor performance. The magnified images in Figure 5 show the fabric under applied strain.

**Figure 6:**
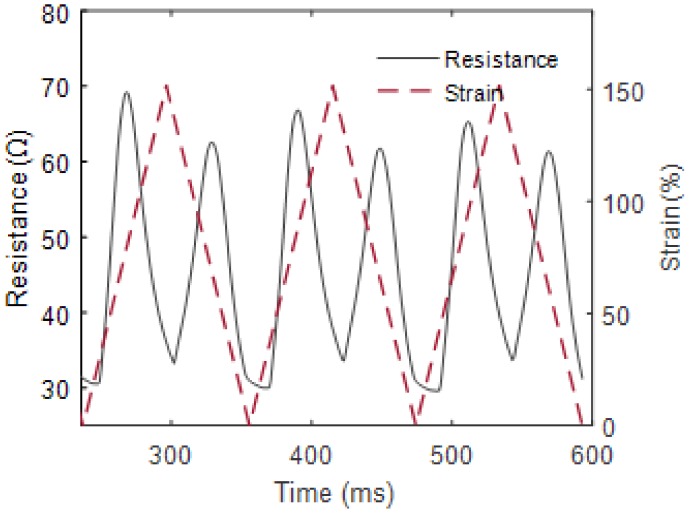
Electrical resistance change of the conductive fabric under strain.

The range of resistance change of the knitted, conductive fabric at 0–50% strains was found to be between 30–40 Ω (Figure 6).

This range of resistance fluctuation contributes to a fast electrical time constant. The time constant of a capacitive sensor is the time required to charge the capacitor through the electrode resistor.

τ = R × C,(2)

where τ is time constant, R is sensor-electrode resistance (Ω), and C is sensor capacitance (F). The time constant for the sensor to charge/discharge is found to be in the order of several nanoseconds.

### 3.3. Experimental Results of Soft Sensor

**Figure 7:**
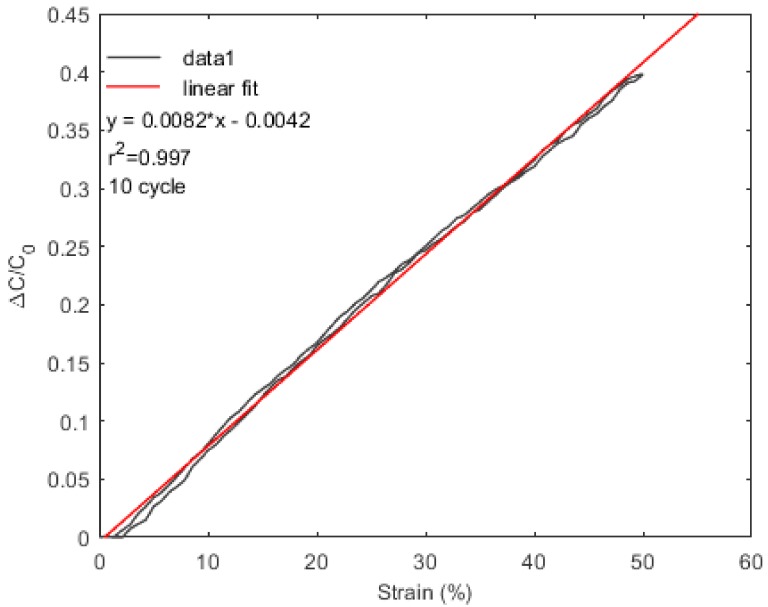
The relative change in capacitance as a function of strain value.

Figure 7 shows the relative change in capacitance, as a function of strain value. The gauge factor (GF) of the sensor is calculated as 0.83. It was reported in previous studies that the GFs of capacitive-based stretch sensors are found to be between 0.7 and 1 [33]. GF can be described as (ΔC/C_0_)/ε, where ΔC is the change in capacitance value, C_0_ is the initial capacitance value, and ε is the strain value. Linearity of the sensor is found to be R^2^ = 0.997. Hysteresis behavior becomes important when the sensor is intended to be used in real-life applications. In this case, the proposed sensor shows negligible hysteresis behavior, which can be explained because the performance of capacitive strain sensors depends on the stable, overlapped parts between electrodes, but not the conductance history of electrodes, unlike resistive sensors, which show large hysteresis behavior [33].

**Figure 8:**
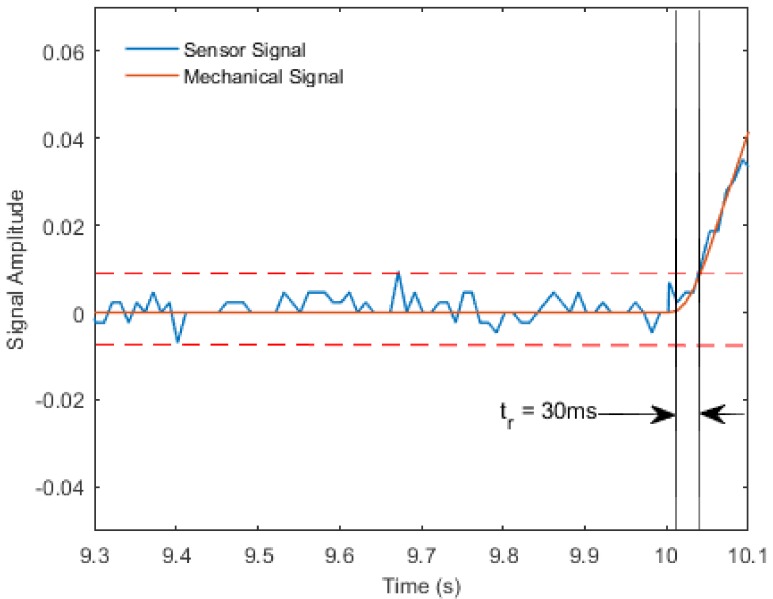
Response time of the sensor.

As shown in Figure 8, the response time of the sensor was calculated as the time span between mechanical stimulation and the point in time when the sensor signal rose three standard deviations above the base signal. The sensor’s delay was found to be 30 ms. Since the frequency of human body motions rarely exceeds 10 Hz, the proposed soft sensor may be considered a suitable device to monitor body motion.

**Figure 9:**
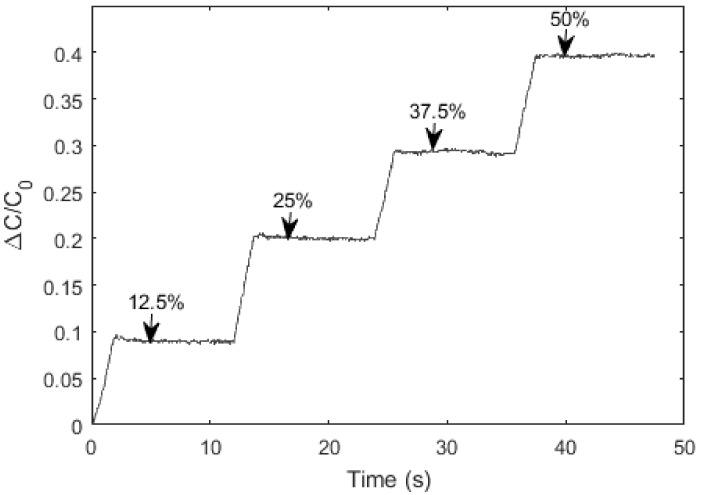
Drift of the sensor under constant strain levels.

In order to identify the resolution of the soft sensor, the noise in the sensor response at the maximum strain level of 50% was measured. Data was recorded when maintaining a sensing bandwidth of 50 Hz. The resolution corresponds to a 95% confidence interval around the measured value. The absolute resolution value was found to be 1.36% of fullscale. Since the stretchable part of the soft sensor is 78 mm, elongations around one millimeter can be successfully monitored. As shown in Figure 9, ε = 0.125, 0.25, 0,375, and 0.5 strain levels were applied to the sensor, and sensors were held for 10 s at these strain levels, in order to observe the drift under static loading. Drift values were calculated as the change in the sensors’ electrical response to a constant strain value. The drift values of the strain sensor were found to be 0.6%, 0.4%, 0.9%, and 0.7% for strain levels of ε = 0.125, 0.25, 0.375, and 0.5 respectively.

**Figure 10:**
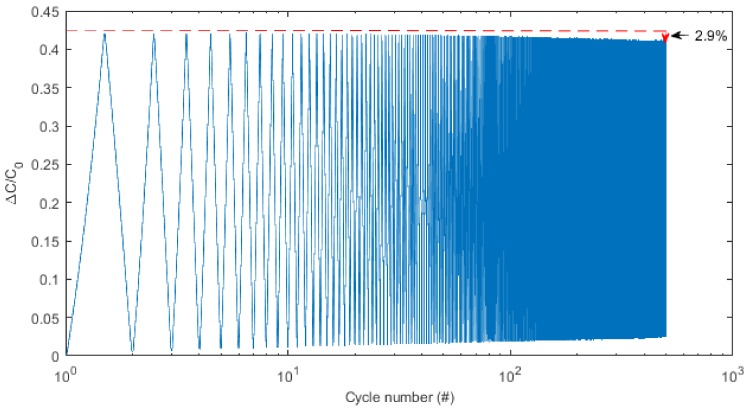
Cyclic test of the sensor under 50% strain.

The durability of the structure is a key property for wearable sensors. As shown in Figure 10, after cyclic stretching and relaxing up to 50% strain for 500 cycles, the ΔC/C_0_ ratio of the soft sensor changed only within 2.9% of its initial value. Hence, the soft-strain sensor proposed here is highly stable under repeated stretching. This could be attributed to the secure conductive network of the knitted fabric structure.

**Figure 11:**
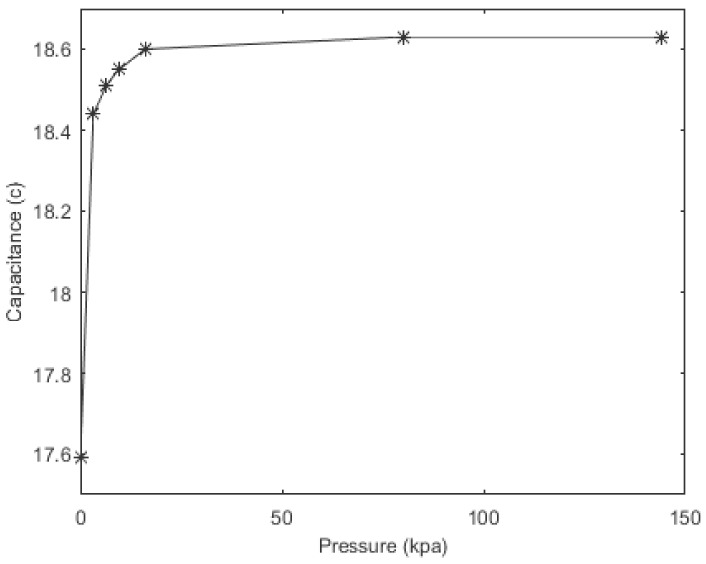
Pressure response of the sensor.

It was estimated that the interdigitated, soft sensor would be insensitive to the applied pressure. In order to show this phenomenon, different pressure ranges were applied to the surface of the sensor. However, when 32 kPa of pressure (using a brass weight) was applied to the sensor, the initial capacitance of the sensor increased by 5.6%. As is explained in previous studies [34], this increase in capacitance stems from the proximity effect of the brass block. Here, permittivity of the conductive electrodes within the structure was increased, thus the capacitance of the soft sensor changed. Thereafter, additional weights were added in order to show the effect of pressure on the soft sensor capacitance values. As shown in Figure 11, since the capacitance values of the sensor barely changed, it can be assumed that the soft sensor is insensitive to applied pressure but is sensitive to applied strain.

**Figure 12:**
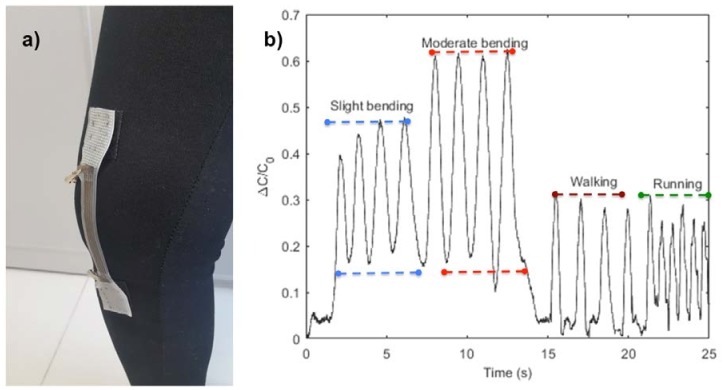
(**a**) Sensor on subject’s knee (**b**) Sensor signals under different knee movements.

To demonstrate the application of the sensor as a wearable device, the strain sensor was used to monitor knee joint movements, as shown in Figure 12a. For monitoring knee movement, the soft sensor was mounted onto an elastic, knitted, fabric tight using Velcro, and worn by the subject. Figure 12b shows the change in capacitance values of the soft strain sensor as the knee joint moves with different strengths and frequencies. Moderate bending of the knee produces a larger change in capacitance value compared with slight bending, which is due to the fact that moderate bending stretches the soft sensor structure more. As seen in Figure 12b, the soft sensor also successfully records walking and running actions. Moreover, the frequencies of walking action and running action were calculated as 0.73 and 1.61, respectively, based on the experimental data.

## 4. Conclusions

Capacitive-based, soft-strain sensors enable scientists to measure human body joint kinematics. Herein, an interdigital-type, capacitive sensor has been developed by combining silicone and a conductive-knit textile. While the conductive-knit electrode creates a secure conductive network, the silicone elastomer helps to protect the dimensional-stability of the structure, and works as a base material of the system. Based on the experimental results, sensors showed highly linear and fast responses. Finally, we showed that this soft sensor system is very convenient for integration into garments for human body joint monitoring. We believe that this type of sensor is a suitable candidate for monitoring human body movements for medical, sports, or entertainment applications. Further experimental work may be performed with alterative materials, i.e., different conductive fabrics and silicone elastomers with different hardness, to tailor the application of specific sensors.

## References

[1] Zhang B.-C., Wang H., Zhao Y., Li F., Ou X.-M., Sun B.-Q., Zhang X.-H. (2016). Large-scale assembly of highly sensitive si-based flexible strain sensors for human motion monitoring. Nanoscale.

[2] Atalay O., Kennon W.R., Demirok E. (2015). Weft-knitted strain sensor for monitoring respiratory rate and its electro-mechanical modeling. Sens. J. IEEE.

[3] Ryu S., Lee P., Chou J.B., Xu R., Zhao R., Hart A.J., Kim S.-G. (2015). Extremely elastic wearable carbon nanotube fiber strain sensor for monitoring of human motion. ACS Nano.

[4] Wang Y., Wang L., Yang T., Li X., Zang X., Zhu M., Wang K., Wu D., Zhu H. (2014). Wearable and highly sensitive graphene strain sensors for human motion monitoring. Adv. Funct. Mater..

[5] Ahmed A., Zhang S.L., Hassan I., Saadatnia Z., Zi Y., Zu J., Wang Z.L. (2017). A washable, stretchable, and self-powered human-machine interfacing triboelectric nanogenerator for wireless communications and soft robotics pressure sensor arrays. Extreme Mech. Lett..

[6] Cho H., Lee H., Kim Y., Kim J. (2017). Design of an optical soft sensor for measuring fingertip force and contact recognition. Int. J. Control Autom. Syst..

[7] Atalay A., Atalay O., Husain M.D., Fernando A., Potluri P. (2017). Piezofilm yarn sensor-integrated knitted fabric for healthcare applications. J. Ind. Text..

[8] Chiu Y.-Y., Lin W.-Y., Wang H.-Y., Huang S.-B., Wu M.-H. (2013). Development of a piezoelectric polyvinylidene fluoride (pvdf) polymer-based sensor patch for simultaneous heartbeat and respiration monitoring. Sens. Actuators A Phys..

[9] Hoffmann T., Eilebrecht B., Leonhardt S. (2011). Respiratory monitoring system on the basis of capacitive textile force sensors. Sens. J. IEEE.

[10] Chen Y., Lu B., Chen Y., Feng X. (2015). Breathable and stretchable temperature sensors inspired by skin. Sci. Rep..

[11] Husain M.D., Naqvi S., Atalay O., Hamdani S.T.A., Kennon R. (2016). Measuring human body temperature through temperature sensing fabric. AATCC J. Res..

[12] Bianchi M., Haschke R., Büscher G., Ciotti S., Carbonaro N., Tognetti A. (2016). A multi-modal sensing glove for human manual-interaction studies. Electronics.

[13] Suh J.-H., Amjadi M., Park I., Yoo H.-J. (2015). Finger motion detection glove toward human-machine interface. Sensors.

[14] Kenny T., Wilson J. (2005). Sensor fundamentals a2. Sensor Technology Handbook.

[15] Atalay O., Kennon W., Husain M. (2013). Textile-based weft knitted strain sensors: Effect of fabric parameters on sensor properties. Sensors.

[16] Bashir T., Ali M., Persson N.-K., Ramamoorthy S.K., Skrifvars M. (2014). Stretch sensing properties of conductive knitted structures of pedot-coated viscose and polyester yarns. Text. Res. J..

[17] Ren J., Wang C., Zhang X., Carey T., Chen K., Yin Y., Torrisi F. (2017). Environmentally-friendly conductive cotton fabric as flexible strain sensor based on hot press reduced graphene oxide. Carbon.

[18] Cochrane C., Lewandowski M., Koncar V. (2010). A flexible strain sensor based on a conductive polymer composite for in situ measurement of parachute canopy deformation. Sensors.

[19] Xu J., Wang D., Fan L., Yuan Y., Wei W., Liu R., Gu S., Xu W. (2015). Fabric electrodes coated with polypyrrole nanorods for flexible supercapacitor application prepared via a reactive self-degraded template. Org. Electron..

[20] Lu Z., Mao C., Zhang H. (2015). Highly conductive graphene-coated silk fabricated via a repeated coating-reduction approach. J. Mater. Chem. C.

[21] Lu M., Xie R., Liu Z., Zhao Z., Xu H., Mao Z. (2016). Enhancement in electrical conductive property of polypyrrole-coated cotton fabrics using cationic surfactant. J. Appl. Polym. Sci..

[22] Kim H.K., Kim M.S., Chun S.Y., Park Y.H., Jeon B.S., Lee J.Y., Hong Y.K., Joo J., Kim S.H. (2003). Characteristics of electrically conducting polymer-coated textiles. Mol. Cryst. Liq. Cryst..

[23] Zhang H., Tao X., Yu T., Wang S. (2006). Conductive knitted fabric as large-strain gauge under high temperature. Sens. Actuators A Phys..

[24] Pacelli M., Loriga G., Paradiso R. Flat knitted sensors for respiration monitoring. Proceedings of the IEEE International Symposium on Industrial Electronics (SIE 2007).

[25] Metcalf C.D., Collie S.R., Cranny A.W., Hallett G., James C., Adams J., Chappell P.H., White N.M., Burridge J.H. Fabric-based strain sensors for measuring movement in wearable telemonitoring applications. Proceedings of the IET Conference on Assisted Living 2009.

[26] Lipomi D.J., Vosgueritchian M., Tee B.C., Hellstrom S.L., Lee J.A., Fox C.H., Bao Z. (2011). Skin-like pressure and strain sensors based on transparent elastic films of carbon nanotubes. Nat. Nanotechnol..

[27] Cai L., Song L., Luan P., Zhang Q., Zhang N., Gao Q., Zhao D., Zhang X., Tu M., Yang F. (2013). Super-stretchable, transparent carbon nanotube-based capacitive strain sensors for human motion detection. Sci. Rep..

[28] Amjadi M., Pichitpajongkit A., Lee S., Ryu S., Park I. (2014). Highly stretchable and sensitive strain sensor based on silver nanowire–elastomer nanocomposite. ACS Nano.

[29] Hu W., Niu X., Zhao R., Pei Q. (2013). Elastomeric transparent capacitive sensors based on an interpenetrating composite of silver nanowires and polyurethane. Appl. Phys. Lett..

[30] Chen T., Xue Y., Roy A.K., Dai L. (2013). Transparent and stretchable high-performance supercapacitors based on wrinkled graphene electrodes. ACS Nano.

[31] Tsouti V., Mitrakos V., Broutas P., Chatzandroulis S. (2016). Modeling and development of a flexible carbon black-based capacitive strain sensor. IEEE Sens. J..

[32] Atalay A., Sanchez V., Atalay O., Vogt D.M., Haufe F., Wood R.J., Walsh C.J. (2017). Batch fabrication of customizable silicone-textile composite capacitive strain sensors for human motion tracking. Adv. Mater. Technol..

[33] Amjadi M., Kyung K.U., Park I., Sitti M. (2016). Stretchable, skin-mountable, and wearable strain sensors and their potential applications: A review. Adv. Funct. Mater..

[34] Kim S.-R., Kim J.-H., Park J.-W. (2017). Wearable and transparent capacitive strain sensor with high sensitivity based on patterned Ag nanowire networks. ACS Appl. Mater. Interfaces.

